# Steroidal Carboxylic Acids from Soft Coral *Paraminabea acronocephala *

**DOI:** 10.3390/md11010136

**Published:** 2013-01-11

**Authors:** Chih-Hua Chao, Yang-Chang Wu, Zhi-Hong Wen, Jyh-Horng Sheu

**Affiliations:** 1 Department of Marine Biotechnology and Resources, National Sun Yat-sen University, Kaohsiung 80424, Taiwan; E-Mails: chaochihhua@hotmail.com (C.-H.C.); wzh@mail.nsysu.edu.tw (Z.-H.W.); 2 Chinese Medicine Research and Development Center, China Medical University Hospital, Taichung 40402, Taiwan; 3 China Medical University, Taichung 40402, Taiwan; 4 Graduate Institute of Integrated Medicine, College of Chinese Medicine, China Medical University, Taichung 40402, Taiwan; E-Mail: yachwu@mail.cmu.edu.tw; 5 Asia-Pacific Ocean Research Center, National Sun Yat-sen University, Kaohsiung 80424, Taiwan

**Keywords:** *Paraminabea acronocephala*, paraminabic acid, soft coral, cytotoxicity, anti-inflammatory activity

## Abstract

Three new steroidal carboxylic acids, paraminabic acids A–C (**1**–**3**) were isolated from a Formosan soft coral *Paraminabea acronocephala*. The structures of these compounds were established by extensive spectroscopic analysis and chemical methods. Application of the PGME method allowed the establishment of the absolute configurations at C-25 and C-24 for **1** and **2**, respectively. Compound **3** showed potent cytotoxicity toward Hep3B, MDA-MB-231, MCF-7, and A-549 cancer cell lines, with IC_50_ values ranging from 2.05 to 2.83 μg/mL. Compounds **2** and **3** were found to inhibit the accumulation of the pro-inflammatory iNOS protein.

## 1. Introduction

**Figure 1:**
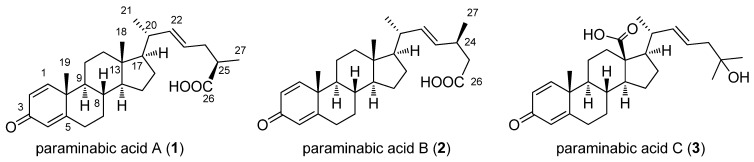
The structures of paraminabic acids A–C (**1**–**3**).

Marine withanolides, with potent pro-inflammatory inducible nitric oxide synthase (iNOS) inhibitory activity, have previously been reported from two species of soft corals, *Paraminabea acronocephala* [1] and *Minabea *sp. [2]*.* These compounds possess a different A-ring structure (1,4-dien-3-one or 4-en-3-one) from those of plant origin [1,2,3]. Our previous chemical investigation of the soft coral *P. acronocephala* led to the isolation of novel withanolides with a 24β,25β-dimethyl-γ-lactone or a 24β,25α-dimethyl-γ-lactone in the steroidal side chain moiety [1]. As part of our continuing search for bioactive, structurally interesting metabolites from this coral, three steroidal carboxylic acids (**1**–**3**) were isolated and their structures were elucidated (Figure 1). The cytotoxicity of compounds **1**–**3** against human liver carcinoma (HepG2 and HepG3), human breast carcinoma (MCF-7 and MDA-MB-231), and human lung carcinoma (A-549) cell lines and the ability of **1**–**3** to inhibit up-regulation of the pro-inflammatory iNOS and COX-2 (cyclooxygenase-2) proteins in LPS (lipopolysaccharide)-stimulated RAW264.7 macrophage cells were also evaluated. 

## 2. Results and Discussion

The ethanolic extract of the soft coral *P. acronocephala* was partitioned between EtOAc and H_2_O to afford the EtOAc-soluble fraction. It was then subjected to silica gel column chromatography. The fractions containing steroids were selected for further study, based on characteristic methyl signals in the ^1^H NMR spectrum. These fractions were subsequently subjected to a series of chromatographic separations to afford three new steroidal carboxylic acids, paraminabic acids A–C (**1**–**3**).

**Figure 2:**
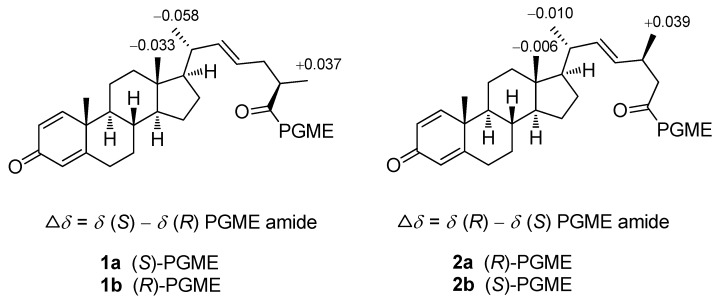
^1^H NMR chemical shift differences of PGME amides of **1** and **2**.

The HRESIMS and ^13^C NMR spectroscopic data of paraminabic acid A (**1**) suggested a molecular formula of C_27_H_38_O_3_, appropriate for nine degrees of unsaturation. The ^13^C NMR and DEPT spectroscopic data (Table 1) displayed 27 carbon signals, including 4 methyls, 7 methylenes, 11 methines, and 5 quaternary carbons. A broad O–H stretching absorption in the region of 3400–2600 cm^−1^ is ascribable to a carboxylic acid, which was evidenced by the carbon resonance at δ_C_ 180.6 (C). The same steroidal nucleus as that of paraminabeolides D and E was deduced for **1** by detailed comparison of their NMR spectroscopic data [1]. The side chain moiety of **1** resembles that of a known steroidal carboxylic acid, (25*S*)-3-oxocholesta-1,4-dien-26-oic acid [4], isolated from the Indonesian soft coral *Minabea *sp. However, **1** varied from (25*S*)-3-oxocholesta-1,4-dien-26-oic acid in the respective side chain. The proton resonances at δ_H_ 5.50 (1H, dt, *J* = 15.6, 6.4 Hz, H-23) and 5.44 (1H, dd, *J* = 15.6, 8.8 Hz, H-22), measured in C_5_D_5_N, were due to the presence of a *trans* C-22/C-23 double bond, which was confirmed by the HMBC correlations from H_3_-21 to C-17, C-20, and C-22. The absolute configuration at C-25 was determined by the application of Kusumi’s method (PGME method) [5,6,7]. The chemical shift differences of (*S*)-PGME amide (**1a**) and (*R*)-PGME amide (**1b**) (Δδ = δ_(*S*)_ − δ_(*R*)_) were summarized in Figure 2 and established the *R *configuration at C-25.

**Table 1:** ^13^C NMR spectroscopic data of compounds **1**−**3**.

Position	1 ^a^, δ_C_, mult.	2 ^a^, δ_C_, mult.	3 ^a^, δ_C_, mult.
1	156.1, CH	156.1, CH	155.9, CH
2	127.4, CH	127.4, CH	127.5, CH
3	186.5, C	186.5, C	186.5, C
4	123.8, CH	123.7, CH	123.8, CH
5	169.5, C	169.6, C	169.3, C
6	32.9, CH_2_	32.9, CH_2_	32.7, CH_2_
7	33.7, CH_2_	33.7, CH_2_	33.4, CH_2_
8	35.5, CH	35.5, CH	37.1, CH
9	52.4, CH	52.4, CH	52.4, CH
10	43.6, C	43.6, C	43.6, C
11	22.8, CH_2_	22.8, CH_2_	24.6, CH_2_
12	39.3, CH_2_	39.3, CH_2_	35.1, CH_2_
13	42.6, C	42.5, C	55.8, C
14	55.6, CH_2_	55.5, CH_2_	55.7, CH_2_
15	24.4, CH_2_	24.3, CH_2_	25.0, CH_2_
16	28.4, CH_2_	28.3, CH_2_	25.3, CH_2_
17	55.5, CH	55.5, CH	55.3, CH
18	12.2, CH_3_	12.2, CH_3_	176.8, C ^b^
19	18.7, CH_3_	18.7, CH_3_	18.7, CH_3_
20	40.0, CH	39.9, CH	38.4, CH
21	20.6, CH_3_	20.6, CH_3_	22.0, CH_3_
22	139.6, CH	136.1, CH	136.7, CH
23	123.8, CH	131.3, CH	124.8, CH
24	36.4, CH_2_	33.7, CH	46.8, CH_2_
25	39.3, CH	41.6, CH_2_^b^	71.7, C
26	180.6, C ^b^	176.9, C ^b^	27.6, CH_3_
27	16.3, CH_3_^b^	20.6, CH_3_	30.5, CH_3_

^a^ Spectra were measured in CDCl_3_ (100 MHz); ^b^ values obtained from the relevant HMBC or HSQC correlation peaks.

Paraminabic acid B (**2**) gave the same molecular formula, C_27_H_38_O_3_, as that of **1**, based on the analysis of the HRESIMS and ^13^C NMR spectroscopic data (Table 1). The NMR spectroscopic data of **2** are similar to those of **1**, but differences were observed in their side chains. The HMBC correlations from H_3_-21 to C-17, C-20, and C-22 allowed the assignment of a C-22/C-23 double bond. A large coupling constant (15.2 Hz, C_5_D_5_N) between H-22 and H-23 suggested the *E* geometry of this double bond. The H-23 signal appeared as a doublet of doublets, revealing that the adjacent carbon (C-24) should be a methine. This might be due to the attachment of a methyl group (δ_H_ 1.03, 3H, d, *J* = 6.4 Hz, H_3_-27) at C-24 (Table 2). This was confirmed by the HMBC correlations from H_3_-27 to C-23, C-24, and C-25 as well as from H_2_-25 to the carboxyl carbon (C-26). The absolute configuration at C-24 of **2** was determined by the application of Kusumi’s method developed for chiral β,β-disubstituted propionic acid derivatives [6,7]. The ^1^H NMR shift differences (Δδ = δ_(*R*)_ − δ_(*S*)_) between the diastereomeric (*R*)- and (*S*)-PGME amides, **2a** and **2b**, respectively, are summarized in Figure 2 and establish the 24*S* configuration for **2**.

**Table 2:** ^1^H NMR spectroscopic data of compounds **1**−**3**.

#	1, δ_H_ (*J* in Hz) ^a^	2, δ_H_ (*J* in Hz) ^a^	3, δ_H_ (*J* in Hz) ^a^
1	7.06, d (10.0)	7.06, d (10.0)	7.03, d (10.0)
2	6.23, dd (10.0, 1.6)	6.24, d (10.0)	6.23, d (10.0)
4	6.07, s	6.07, s	6.07, s
6	a: 2.45, m	a: 2.46, td (13.6, 4.4)	a: 2.46, td (13.4, 4.4)
	b: 2.35, m	b: 2.35, m	b: 2.35, m
7	a: 1.93, m	a: 1.93, m	a: 2.04, m
	b: 1.02, m	b: 1.02, m	b: 1.07, m
8	1.60, m	1.59, m	1.69, m
9	1.04, m	1.03, m	1.10, m
11	1.67, m	1.67, m	1.85, m
			1.69, m
12	a: 2.00, m	a: 1.99, m	a: 2.66, br d (14.0)
	b: 1.18, m	b: 1.17, m	b: 1.03, m
14	1.12, m	1.11, m	1.30, m
15	a: 1.55, m	a: 1.52, m	a: 1.91, m
	b: 1.08, m	b: 1.02, m	b: 1.66, m
16	a: 1.65, m	a: 1.62, m	a: 1.78, m
	b: 1.22, m	b: 1.20, m	b: 1.70, m
17	0.99, m	0.99, m	1.62, m
18	0.74, s	0.74, s	
19	1.23, s	1.23, s	1.15, s
20	2.03, m	2.00, m	2.36, m
21	0.99, d (6.8)	0.98, d (6.4)	1.05, d (6.4)
22	5.27–5.30 ^b^	5.23–5.26 ^b^	5.39 dd (15.2, 8.8)
23	5.27–5.30 ^b^	5.23–5.26 ^b^	5.48, ddd (15.2, 8.8, 5.2)
24	a: 2.33, m	2.59, m	2.18, dd (14.0, 5.2)
	b: 2.10, m		2.11, dd (14.0, 8.8)
25	2.49, m	2.30, d (7.2)	
26			1.25, s
27	1.15, d (7.2)	1.03, d (6.4)	1.25, s

^a^ Spectra were measured in CDCl_3_ (400 MHz); ^b^ overlapped signals.

The HRESIMS and ^13^C NMR spectroscopic data of paraminabic acid C (**3**) established a molecular formula of C_27_H_38_O_4_ and nine degrees of unsaturation. The IR absorptions at 3419 and 1714 cm^−1^ suggested the presence of hydroxy and carbonyl groups, respectively. Both ^1^H and ^13^C NMR spectra of **3** lacked signals of the angular methyl group, which might be replaced by a carboxylic acid according to the carbon signal at δ_C_ 176.8 (C) (Table 1). The carboxylic acid attached at C-13 was further confirmed by the HMBC correlations from both H_2_-12 and H-17 to C-18. The *trans* C-22/C-23 double bond was deduced by the HMBC correlations from H_3_-21 to C-17, C-20, and C-22 as well as *J* value (15.2 Hz) (Table 2) between H-22 and H-23. In addition, the downfield-shifted quaternary carbon at δ_C_ 69.9 was ascribable to a hydroxy group attached at C-25, which was correlated by H_2_-24, H_3_-26, and H_3_-27 in the HMBC spectrum. 

**Figure 3:**
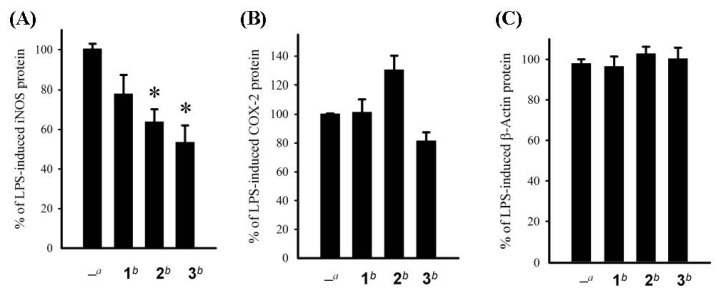
Effect of compounds **1**–**3** at 10 μM on the LPS-induced pro-inflammatory iNOS and on COX-2 protein expression of RAW264.7 macrophage cells by immunoblot analysis (**A**) Quantification of immunoblots of iNOS; (**B**) Quantification of immunoblots of COX-2. The values are means ± SEM (*n* = 6). The relative intensity of the LPS alone stimulated group was taken as 100%. * Significantly different from LPS alone stimulated group (* *P* < 0.05). ^a^ Stimulated with LPS. ^b^ Stimulated with LPS in the presence of **1**–**3** (10 μM); (**C**) Quantification of immunoblots of β-actin.

The cytotoxicity of compounds **1**–**3** against HepG2, Hep3B, MDA-MB-231, MCF-7, and A-549 cancer cells was studied and shown in Table 3. Compound **3** showed potent cytotoxicity toward Hep3B, MDA-MB-231, MCF-7, and A-549 cancer cell lines, with IC_50_ values ranging from 2.05 to 2.83 μg/mL. We also investigated the inhibition of these compounds toward LPS-induced pro-inflammatory protein (iNOS and COX-2) expression in RAW264.7 macrophage cells by Western blot analysis. At a concentration of 10 μM, compounds **2 **and **3** reduced the levels of iNOS to 63.9 ± 6.3% and 53.5 ± 8.6%, respectively; whereas, compound **2** enhanced the expression of COX-2 (130.5 ± 9.8%) in comparison with those of control cells stimulated with LPS only (100% for both iNOS and COX-2). Also, compound **3** could inhibit the expression of iNOS protein but did not induce cytotoxicity in macrophage cells as determined through internal control β-actin expression, as shown in Figure 3. These results indicate that **3** possesses moderate anti-inflammatory activity and potent cytotoxicity, and might be useful for further medicinal study.

**Table 3:** Cytotoxicity data of compounds **1**–**3**.

Compound		Cell lines IC_50_ (μg/mL)			
	Hep G2	Hep 3B	MDA-MB-231	MCF-7	A549
**1**	15.21	–	19.66	–	–
**2**	19.77	–	–	–	–
**3**	13.57	2.83	2.25	2.23	2.05
doxorubicin	0.31	0.40	1.32	0.68	1.33

(–): Compound is considered inactive with IC_50_ > 20 μg/mL.

## 3. Experimental Section

### 3.1. General Experimental Procedures

Optical rotations were determined with a JASCO P1020 digital polarimeter. The IR spectra were obtained on a JASCO FT/IR-4100 spectrophotometer. The NMR spectra were recorded on a Bruker AVANCE 300 FT-NMR (or Varian MR-400 NMR) instrument at 300 MHz (or 400 MHz) for ^1^H (referenced to TMS for both CDCl_3_ and C_5_D_5_N) and 75 MHz (or 100/125 MHz) for ^13^C (referenced to δ_C_ 77.0 for CDCl_3_ and to internal TMS for C_5_D_5_N). ESIMS were recorded by ESI FT-MS on a Bruker APEX II mass spectrometer. Silica gel 60 (230−400 mesh, Merck, Darmstadt, Germany) and LiChroprep RP-18 (Merck, 40–63 μm) were used for column chromatography. Precoated silica gel plates (Kieselgel 60 F254, 0.25 mm, Merck, Darmstadt, Germany) and precoated RP-18 F254S plates (Merck, Darmstadt, Germany) were used for TLC analyses. High-performance liquid chromatography was performed on a Hitachi L-7100 pump equipped with a Hitachi L-7400 UV detector at 210 nm and a semi-preparative reversed-phase column (Hibar Purospher RP-18e, 5 μm, 250 × 10 mm, Merck, Darmstadt, Germany).

### 3.2. Animal Material

The soft coral *P. acronocephala* was collected by scuba divers, off the western coast of Pingtung county, in May 2009, at a depth of 10 m, and was stored in a freezer until being extracted. This soft coral was identified by Prof. Chang-Fong Dai, Institute of Oceanography, National Taiwan University. A voucher specimen (specimen No. 200905PA) was deposited in the Department of Marine Biotechnology and Resources, National Sun Yat-sen University. 

### 3.3. Extraction and Isolation

The soft coral *P. acronocephala* (3.8 kg fresh wt) was collected and freeze-dried. The freeze-dried material was minced and extracted exhaustively with EtOH (6 × 2 L). The organic extract was concentrated to an aqueous suspension and was further partitioned between EtOAc and water. The EtOAc extract (30 g) was fractionated by open column chromatography on silica gel using *n*-hexane–EtOAc and EtOAc–MeOH mixtures of increasing polarity to yield 28 fractions. Fraction 21 (3.6 g), eluted with *n*-hexane–EtOAc (1:6), was further separated by silica gel column chromatography with gradient elution (*n*-hexane-acetone, 5:1 to 2:1) to yield eight subfractions (21A to 21H). Subfraction 21D was fractionated by RP-18 open column (MeOH–H_2_O, 50% to 100%) to afford six subfractions (21D1 to 21D6). Compounds **1** (1.9 mg) and **2** (2.6 mg) were obtained from subfraction 21D5 using RP-18 HPLC (MeOH–H_2_O, gradient 75% to 85%). Compound **3** (5.1 mg) was obtained from fraction 25 (0.85 g) using repeatedly column chromatography over silica gel (*n*-hexane–EtOAc, 1:3 to 0:1) and RP-18 gel (MeOH–H_2_O, 50% to 100%), and subsequently separated by RP-18 HPLC (MeOH–H_2_O, gradient, 65%–75%). 

Paraminabic acid A (**1**): amorphous solid; [α]^24^_D_ +13 (*c* 0.09, CHCl_3_); IR (KBr) ν_max_ 3400–2600 (br), 2933, 2868, 2853, 1718, 1662, 1615, 1602, 1457, 1406, 1375, 1292, 1241 cm^−1^; ^13^C NMR and ^1^H NMR data, see Table 1 and Table 2; Selected ^1^H NMR (C_5_D_5_N, 400 MHz) of **1**: δ 7.01 (1H, d, *J* = 10.0 Hz, H-1), 6.42 (1H, dd, *J* = 10.0, 2.0 Hz, H-2), 6.26 (1H, s, H-4), 5.50 (1H, dt, *J* = 15.6, 6.4 Hz, H-23), 5.44 (1H, dd, *J* = 15.6, 8.8 Hz, H-22), 2.78 (1H, m, H-25), 2.65 (1H, m, H-24a), 2.40 (1H, m, H-24b), 1.38 (3H, d, *J* = 6.8 Hz, H_3_-27), 1.10 (3H, s, H_3_-19), 1.03 (3H, d, *J* = 6.4 Hz, H_3_-21), 0.67 (3H, s, H_3_-18); ESIMS *m/z* 433 [M + Na]^+^; HRESIMS *m/z* 433.2715 [M + Na]^+^ (calcd for C_27_H_38_O_3_Na, 433.2718).

Paraminabic acid B (**2**): amorphous solid; [α]^24^_D_ +60 (*c* 0.16, CHCl_3_); IR (KBr) ν_max_ 3400–2600 (br), 2934, 2868, 1718, 1662, 1617, 1601, 1455, 1405, 1375, 1292, 1241 cm^−1^; ^13^C NMR and ^1^H NMR data, see Table 1 and Table 2; ^1^H NMR (C_5_D_5_N, 400 MHz) of **2**: δ 7.01 (1H, d, *J* = 10.0 Hz, H-1), 6.42 (1H, dd, *J* = 10.0, 1.6 Hz, H-2), 6.27 (1H, s, H-4), 5.51 (1H, dd, *J* = 15.2, 7.2 Hz, H-23), 5.41 (1H, dd, *J* = 15.2, 8.4 Hz, H-22), 2.97 (1H, m, H-24), 2.63 (1H, dd, *J* = 14.8, 7.6 Hz, H-25a), 2.55 (1H, dd, *J* = 14.8, 7.2 Hz, H-25b), 2.31 (1H, td, *J* = 13.6, 4.4 Hz, H-6a), 2.18 (1H, dt, *J* = 13.6, 2.4 Hz, H-6b), 2.04 (1H, m, H-20), 1.90 (1H, dt, *J* = 12.4, 3.2 Hz, H-12a), 1.71 (1H, m, H-16a), 1.70 (1H, m, H-7a), 1.54 (2H, m, H_2_-11), 1.42 (1H, m, H-8), 1.39 (1H, m, H-15a), 1.25 (1H, m, H-16b), 1.21 (3H, d, *J* = 6.8 Hz, H_3_-27), 1.09 (3H, s, H_3_-19), 1.08 (1H, m, H-17), 1.06 (1H, m, H-12b), 1.04 (3H, d, *J* = 6.0 Hz, H_3_-21), 0.99 (1H, m, H-15b), 0.88 (1H, m, H-9), 0.83 (1H, m, H-14), 0.82 (1H, m, H-7b), 0.67 (3H, s, H_3_-18); ^13^C NMR (C_5_D_5_N, 100 MHz) of **2**: δ 185.9 (C, C-3), 175.2 (C, C-26), 169.2 (C, C-5), 156.0 (CH, C-1), 135.5 (CH, C-22), 132.7 (CH, C-23), 127.7 (CH, C-2), 124.0 (CH, C-4), 55.9 (CH, C-17), 55.7 (CH, C-14), 52.6 (CH, C-9), 43.7 (C, C-10), 43.1 (CH_2_, C-25), 42.7 (C, C-13), 40.3 (CH, C-20), 39.6 (CH_2_, C-12), 35.4 (CH, C-8), 34.1 (CH, C-24), 33.8 (CH_2_, C-7), 32.9 (CH_2_, C-6), 28.7 (CH_2_, C-16), 24.4 (CH_2_, C-15), 22.9 (CH_2_, C-11), 20.9 (CH_3_, C-21), 20.8 (CH_3_, C-27), 18.7 (CH_3_, C-19), 12.3 (CH_3_, C-18); ESIMS *m/z* 433 [M + Na]^+^; HRESIMS *m/z* 433.2715 [M + Na]^+^ (calcd for C_27_H_38_O_3_Na, 433.2718).

Paraminabic acid C (**3**): amorphous solid; [α]^24^_D_ +43 (*c* 0.09, CHCl_3_); IR (KBr) ν_max_ 3400–2600 (br), 3419, 2967, 2936, 2870, 1714, 1660, 1616, 1599, 1456, 1442, 1375, 1295, 1240, 1161cm^−1^; ^13^C NMR and ^1^H NMR data, see Table 1 and Table 2; ^1^H NMR (C_5_D_5_N, 400 MHz) of **3**: δ 7.01 (1H, d, *J* = 10.0 Hz, H-1), 6.41 (1H, dd, *J* = 10.0, 2.0 Hz, H-2), 6.25 (1H, s, H-4), 5.73 (1H, dt, *J* = 15.2, 7.4 Hz, H-23), 5.49 (1H, dd, *J* = 15.2, 8.4 Hz, H-22), 3.00 (1H, br d, *J* = 12.4 Hz, H-12a), 2.47 (1H, m, H-20), 2.41 (2H, m, H_2_-24), 2.25 (1H, m, H-15a), 2.22 (1H, m, H-6a), 2.17 (1H, m, H-6b), 2.02 (1H, m, H-8), 1.98 (1H, m, H-16a), 1.92 (2H, m, H-7a and H-11a), 1.90 (1H, m, H-16b), 1.84 (1H, m, H-11b), 1.64 (1H, m, H-15b), 1.60 (1H, m, H-17), 1.40 (3H, s, H_3_-26), 1.39 (3H, d, *J* = 6.4 Hz, H_3_-21), 1.39 (3H, s, H_3_-27), 1.38 (1H, m, H-14), 1.22 (1H, m, H-12b), 1.06 (1H, m, H-9), 1.00 (3H, s, H_3_-19), 0.80 (1H, m, H-7b); ^13^C NMR (C_5_D_5_N, 100 MHz) of **3**: δ 185.9 (C, C-3), 176.9 (C, C-18), 169.1 (C, C-5), 155.9 (CH, C-1), 139.3 (CH, C-22), 127.7 (CH, C-2), 125.3 (CH, C-23), 124.2 (CH, C-4), 69.9 (CH, C-25), 57.1 (C, C-13), 56.4 (CH, C-14), 56.0 (CH, C-17), 52.6 (CH, C-9), 48.2 (CH_2_, C-24), 43.7 (C, C-10), 42.0 (CH, C-20), 37.5 (CH, C-8), 37.2 (CH_2_, C-12), 33.8 (CH_2_, C-7), 32.8 (CH_2_, C-6), 30.5 (CH_2_, C-16), 29.9 (CH_3_, C-27), 29.7 (CH_3_, C-26), 25.5 (CH_2_, C-15), 25.2 (CH_2_, C-11), 21.0 (CH_3_, C-21), 18.7 (CH_3_, C-19); ESIMS *m/z* 449 [M + Na]^+^; HRESIMS *m/z* 449.2666 [M + Na]^+^ (calcd for C_27_H_38_O_4_Na, 449.2668).

### 3.4. Preparation of (*S*) and (*R*)-PGME amides of ***1*** and ***2***

To a stirred solution of compound **1** (0.5 mg) and (*S*)-PGME (2 mg) in a 1 mL mixture of CHCl_3_–DMF (10:1) were successively added DMAP (2 mg) and 4-DMAP·HCl (2 mg) [5]. After the mixture was stirred at 0 °C for 5 min, EDC·HCl (2 mg) was added. The reaction mixture was then moved to a refrigerator at 4 °C for overnight. The mixture was then stirred at room temperature for 3 h. Subsequently, ethyl acetate was added, and the resulting solution was successively washed with 5% HCl, saturated NaHCO_3_ (aq), and brine. The organic layer was dried over anhydrous Na_2_SO_4_ and concentrated to give a residue, which was chromatographed on silica gel using *n*-hexane–EtOAc (5:1) as eluent to afford the (*S*)-PGME amide (**1a**) (0.3 mg). The same procedure was used to prepare the (*R*)-PGME amide (**1b**) (0.3 mg from 0.5 mg of **1**) with (*R*)-PGME. Selective ^1^H NMR (CDCl_3_, 300 MHz) of **1a**: δ 7.343 (5H, br s, Ph), 7.049 (1H, d, *J* = 10.2 Hz, H-1), 6.384 (1H, d, *J* = 7.0 Hz, N*H*), 6.228 (1H, d, *J* = 10.2 Hz, H-2), 6.068 (1H, s, H-4), 5.586 (1H, d, *J* = 7.0 Hz, C*H*-N), 5.207 (2H, overlapped, H-22 and H-23), 3.730 (3H, s, O*Me*), 1.225 (3H, s, H_3_-19), 1.143 (3H, d, *J* = 6.3 Hz, H_3_-27), 0.911 (3H, d, *J* = 6.4 Hz, H_3_-21), 0.711 (3H, s, H_3_-18); selective ^1^H NMR (CDCl_3_, 300 MHz) of **1b**: *δ* 7.345 (5H, br s, Ph), 7.052 (1H, d, *J* = 9.7 Hz, H-1), 6.405 (1H, d, *J* = 7.4 Hz, N*H*), 6.226 (1H, d, *J* = 9.7 Hz, H-2), 6.066 (1H, s, H-4), 5.568 (1H, d, *J* = 7.4 Hz, C*H*-N), 5.286 (2H, overlapped, H-22 and H-23), 3.726 (3H, s, O*Me*), 1.229 (3H, s, H_3_-19), 1.106 (3H, d, *J* = 6.0 Hz, H_3_-27), 0.969 (3H, d, *J* = 6.5 Hz, H_3_-21), 0.744 (3H, s, H_3_-18). The same procedure was applied on **2** (0.5 mg) to prepare the (*R*)-PGME amide **2a** (0.4 mg) and the (*S*)-PGME amide **2a** (0.4 mg from 0.5 mg of **2**). Selective ^1^H NMR (CDCl_3_, 300 MHz) of **2b**: δ 7.357 (5H, br s, Ph), 7.050 (1H, d, *J* = 10.1 Hz, H-1), 6.362 (1H, d, *J* = 7.4 Hz, N*H*), 6.224 (1H, d, *J* = 10.1 Hz, H-2), 6.067 (1H, s, H-4), 5.597 (1H, d, *J* = 7.4 Hz, C*H*-N), 5.241 (2H, overlapped, H-22 and H-23), 3.727 (3H, s, O*Me*), 1.225 (3H, s, H_3_-19), 1.016 (3H, d, *J* = 6.6 Hz, H_3_-27), 0.947 (3H, d, *J* = 6.5 Hz, H_3_-21), 0.716 (3H, s, H_3_-18); selective ^1^H NMR (CDCl_3_, 300 MHz) of **2a**: δ 7.341 (5H, br s, Ph), 7.051 (1H, d, *J* = 10.2 Hz, H-1), 6.455 (1H, d, *J* = 6.9 Hz, N*H*), 6.223 (1H, d, *J* = 10.2 Hz, H-2), 6.066 (1H, s, H-4), 5.579 (1H, d, *J* = 6.9 Hz, C*H*-N), 5.238 (2H, overlapped, H-22 and H-23), 3.722 (3H, s, O*Me*), 1.226 (3H, s, H_3_-19), 0.977 (3H, d, *J* = 6.2 Hz, H_3_-27), 0.957 (3H, d, *J* = 6.0 Hz, H_3_-21), 0.722 (3H, s, H_3_-18). It has to be noted that the chemical shifts of H-22 and H-23 in both PGME amides of **1** and **2** were overlapped seriously, that might interfere the correct assignment of the corresponding protons. Fortunately, we afford the Δδ values of the H_3_-21 and H_3_-18 of (*S*) and (*R*)-PGME amides of both **1** and **2** which could be used for configuration assignment of C-25 in **1** and C-24 in **2**, respectively.

### 3.5. Cytotoxicity Testing

Cell lines were purchased from the American Type Culture Collection (ATCC). Compounds were assayed for cytotoxicity against human liver carcinoma (HepG2 and HepG3), human breast carcinoma (MCF-7 and MDA-MB-231), and human lung carcinoma (A-549) cells using the 3-(4,5-dimethylthiazol-2-yl)-2,5-diphenyltetrazolium bromide (MTT) method [8]. Freshly trypsinized cell suspensions were seeded in 96-well microtiter plates at densities of 5000–10,000 cells per well with tested compounds added from DMSO-diluted stock. After 3 days in culture, attached cells were incubated with MTT (0.5 mg/mL, 1 h) and subsequently dissolved in DMSO. The absorbency at 550 nm was then measured using a microplate reader. The IC_50_ is the concentration of agent that reduced cell growth by 50% under the experimental conditions. 

### 3.6. *In Vitro* Anti-Inflammatory Assay

Macrophage (RAW264.7) cell was purchased from ATCC. *In vitro* anti-inflammatory activities of tested compounds were measured by examining the inhibition of lipopolysaccharide (LPS) induced upregulation of iNOS and COX-2 proteins in macrophage cells using Western blotting analysis [9,10].

## 4. Conclusions

Our previous investigation on *P. acronocephala* has successfully discovered marine withanolides with potent anti-inflammatory activity. In this study, we reported three steroidal carboxylic acids, of which **3** exhibited potent cytotoxicity toward Hep3B, MDA-MB-231, MCF-7, and A-549 cancer cell lines. Compound **2**, the second member of 27-norergostan-26-oic acid obtained from nature [11,12], was isolated from the soft coral for the first time. Our present investigation demonstrated that the soft coral, *P. acronocephala*, is a useful source for the discovery of bioactive substances.
